# Root Canal Filling Quality Comparison of a Premixed Calcium Silicate Endodontic Sealer and Different Carrier-Based Obturation Systems

**DOI:** 10.3390/jcm10061271

**Published:** 2021-03-18

**Authors:** Ruth Pérez-Alfayate, Montse Mercade, Juan Algar-Pinilla, Rafael Cisneros-Cabello, Federico Foschi, Stephen Cohen

**Affiliations:** 1 Department of Endodontics, Universidad Europea de Madrid, 28045 Madrid, Spain; ruthip.alfayate@gmail.com (R.P.-A.); juan.algar2@universidadeuropea.es (J.A.-P.); rafael.cisneros@universidadeuropea.es (R.C.-C.); 2 Department of Dentistry, Universitat de Barcelona, 08907 Barcelona, Spain; montsemercade@ub.edu; 3 Biomedical Research Institute (IDIBELL), 08907 Barcelona, Spain; 4 Department of Endodontics, Faculty of Dentistry, Oral & Craniofacial Sciences, Guy’s Dental Hospital, London SE1 9RT, UK; 5 Department of Therapeutic Dentistry, I. M. Sechenov First Moscow State Medical University, 119146 Moscow, Russia; 6 Peninsula School of Medicine and Dentistry, Plymouth University, Plymouth PL4 8AA, UK; 7 Stephen Cohen Endodontics Clinic, San Francisco, CA 94108, USA; scohen@cohenendodontics.com

**Keywords:** bioceramic sealer, confocal microscopy, cross-linked gutta-percha, curved root canals, sealer placement

## Abstract

Background: The number of voids within the root canal obturation is a relevant parameter to describe the quality of the technique, as well as to predict long-term prognosis. The aim of this study was to evaluate the quality of root canal obturation in curved root canals filled with Thermafil, GuttaCore, GuttaFusion and lateral compaction with AH Plus and EndoSequence BC sealer, by means of percentage of gutta-percha and sealer filled area. Methods: 200 curved canals of mandibular molars were divided in five groups (*n* = 40). Each specimen was evaluated at 3, 6 and 9 mm from the apex. A total of 600 samples were analyzed. Obturation was performed with either Thermafil (TH), GuttaCore (GC), GuttaFusion (GF) or lateral compaction (LC), using AH Plus as sealer. In another group EndoSequence BC sealer (BC) was used. Subgroups (*n* = 20) were made depending on the use of sonic activation during the placement of the sealer. The percentage of total obturation material was analyzed as an indirect measurement of percentage of voids. Results: GF showed a better performance at the apical level, while GC showed the higher percentage of total obturation at the coronal area. No differences were observed for the activation of sealers in any of the groups. Statistical analysis was performed by using two-way ANOVA. Statistical significance was set at CI: 95% (*p* < 0.05). Conclusions: Sonic activation of sealers did not show any benefit to avoid the presence of voids within obturation of curved canals. Following the manufacturer recommendations, we found that TH did not show benefits when applied to curved canals.

## 1. Introduction

Since the main cause of pulpal and periapical disease is the presence of microorganisms and their bioproducts, the outcome of endodontic treatment depends upon efficient cleaning and shaping of the root canals [1]. Given that obtaining total disinfection of the root canal system is not completely achievable [2], it is critical to aim for a three-dimensional root canal filling with a tight seal impermeable to fluids, to prevent oral and apical microleakage, hence minimizing the chances of relapsing endodontic infections [1]. For this purpose, many techniques have been widely studied and described in the literature, such as lateral compaction [3] or carrier-based Thermafil (Dentsply Sirona, Ballaigues, Switzerland) [4]. Thermafil obturation system was affected by some limitations related to re-treatment and post-placement. For these reasons, GuttaCore (Dentsply Sirona, Ballaigues, Switzerland) and GuttaFusion (VDW, Munich, Germany) have been introduced to the market, in an attempt to overcome the limitations of their predecessor, and with the potential to improve the clinical applicability. In a previous study [5], the quality of this two cross-linked carrier-based root canal obturation systems in curved root canals was evaluated with and without sonic activation of the sealer. The observations suggested that both systems were able to produce a homogenous filling when compared to lateral compaction (gold standard) [5]. On the other hand, bioceramic-based materials (containing calcium silicate and/or calcium phosphate) have attracted considerable attention because of their physical and biological properties, namely their alkaline pH, chemical stability within the biological environment, lack of toxicity, biocompatibility and absence of shrinkage [6,7,8]. EndoSequence BC sealer (Brasseler USA, Savannah, GA, USA) is a premixed tricalcium silicate–based root canal sealer that requires moisture from the root dentin to hydrate and set and can be considered as an obturation system by itself [7,9], producing a hydraulic compaction similar to that achieved by carrier-based systems.

The number of voids is a relevant descriptor for assessing the quality of root canal obturation [10,11], and sectioning methods have been suggested as the most appropriate for this purpose [10].

In this study, the null hypothesis tested was that the quality of root canal filling in curved canals of mandibular molars was independent from the type of technique employed. Thus, the aim was two-fold: to compare the quality of root canal obturation of Thermafil, EndoSequence BC sealer and the results obtained for GuttaCore, GuttaFusion and lateral compaction by means of percentage of gutta-percha filled areas (PGFA), percentage of sealer filled areas (PSFA) and voids through coronal, middle and apical histological slices of curved roots canals observed with confocal laser scanning microscopy (CLSM); and to evaluate the influence of cement activation on the sealing ability of the systems used in this study.

## 2. Materials and Methods

The approval of the ethical and research committee of Universidad Europea de Madrid was obtained before performing the study (CIPI/006/14). An in vitro randomized trial was designed based on our previous publication [5] and following the same methodology in which three obturation systems were compared (GuttaCore (GC), GuttaFusion (GF) and lateral condensation (LC)) by means of PGFA, PSFA and voids through coronal, middle and apical histological slices of 120 mandibular curved root canals corresponding to 360 histological slices and 720 image analysis: 360 acquired through CLSM and epi-fluorescence method (Leica TCS SP5; Leica Microsystems GmbH, Mannheim, Germany) and 360 with an operating microscope at 30× as referencing images (Zeiss, Zeiss OPMI pico, Jena, Germany). Briefly, pre-selected teeth were radiographed and then selected according to the following morphological parameters: intact tooth with fully formed apices, mesial root with type III of the Weine configuration and canal with a total length of 14 ± 1 mm from canal orifice to apical foramen. Primary root curvature ranged from 20° to 89°, according to the Pruett method [12,13].

**Figure 1:**
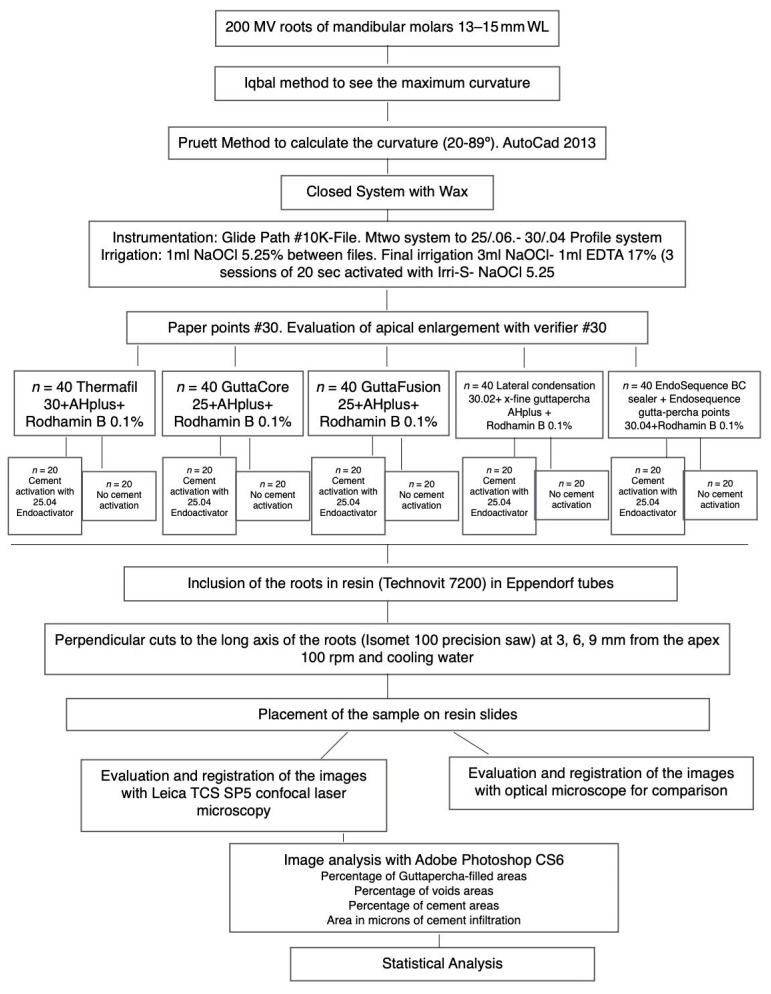
Flowchart of the methodology.

**Figure 2:**
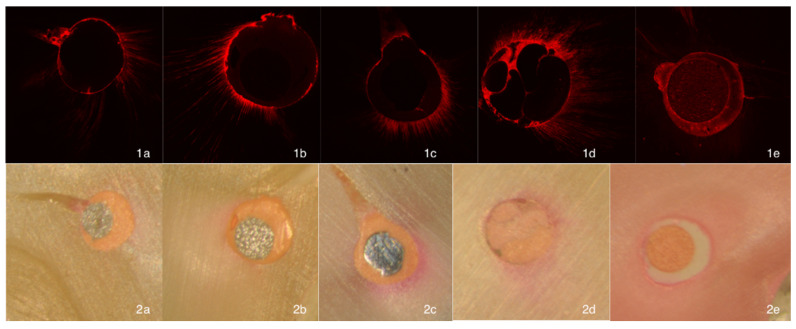
Representative confocal laser scanning microscopic images (1) and corresponding optic images (2) from each experimental group: (**a**) GuttaFusion, (**b**) GuttaCore, (**c**) Thermafil, (**d**) Lateral Compaction and (**e**) EndoSequence BC sealer.

In this new analysis, we included Thermafil (TH) and EndoSequence BC (BC). Thus, 80 more specimens were added, reaching a total sample size of 240 and 480 images to analyze. Each group was dichotomized following the placement technique of the sealer (with sonic activation or without sonic activation). The design of the experimental groups and preparation of the canals was identical to our previous study [5]. A graphic summary of the methodology is presented in Figure 1. For the obturation of the new specimens, rhodamine-B was weighted with a laboratory micro weighing scale and added to AH Plus (Dentsply DeTrey Gmbh, Konstanz, Germany) and EndoSequence BC sealers (0.1%), to facilitate fluorescence with confocal microscopy. An insulin syringe was used to deliver 0.05 mL of cement into the canal. In the groups with sealer activation, the cement was distributed with an “in-and-out” movement for 20 s, using an EndoActivator tip size #25.04 (Dentsply Maillefer) placed 2 mm from working length. Size #30 obturators were selected for Thermafil, according to manufacturer’s recommendation; and for EndoSequence BC sealer group, an EndoSequence BC point size #30.04 was placed to the working length, likewise, following manufacturer instructions. Sectioning of the specimens, CLMS and operatory microscopy analysis were done by following the same parameters as previously described [5] (Figure 2).

### Statistical Analysis

A statistics software (Stata/IC 13.1 for Windows) was used to analyze the data. The graphics were obtained with the IBM SPSS Statistics20 software. The variables were described based on the mean and the standard deviation. The filling groups were evaluated by means of PGFA and stratified by activation. A two-way variance ANOVA test was applied in each area of the section in order to evaluate the interaction between the obturation system used and the activation. When the interaction was statistically significant, a one-way ANOVA test was conducted. When the interaction was non-significant, it was analyzed in the same way without stratification dependent on the activation. To compare the systems in pairs, Sidak correction for multiple comparisons was used.

Since the percentage of voids is inversely related to the total obturation material within the canal, this parameter was used to describe the results.

## 3. Results

The percentage of total obturation material was evaluated at the apical area, middle area and coronal area, meaning at 3, 6 and 9 mm from the apex, respectively. All groups were compared with each other (Table 1).

No differences were observed when comparing activation of sealer in any of the groups of study or in the three different areas.

**Figure 3:**
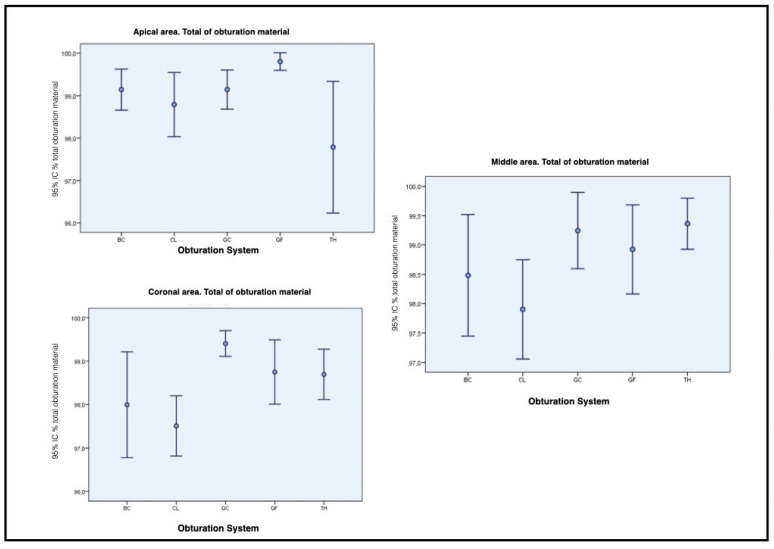
Boxplots representing the percentage of total obturation material in the apical, middle and coronal area.

**Figure 4:**
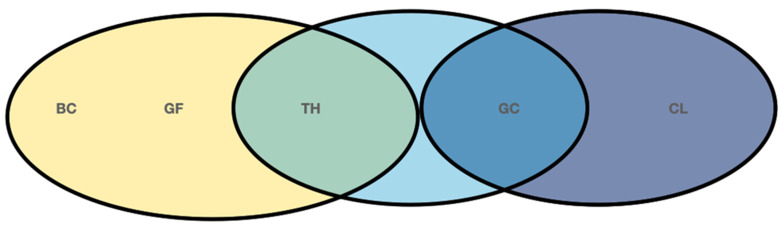
Graphic representation of the differences found between obturation systems. No differences found between systems within the same circle. BC, EndoSequence BC sealer; GF, GuttaFusion, TH Thermafil; GC, GuttaCore; CL, cold lateral compaction.

Statistical differences in the percentage of obturation material (gutta-percha plus sealer) between TH and GF at the apical area, at 3 mm from the apex, were observed. TH filled a mean of 97.6% (SD 4.5) of the cavity, while GF filled the 99.9 % of it (SD: 0.9) (CI: 95%; *p* = 0.006). At the 6 mm section, no statistical differences at the 95% confidence interval were found, when comparing the different treatments. Nevertheless, statistical differences were found between CL and GC treatment groups at the coronal area: 97.6%, SD:1.9 vs. 99.5%, SD:1.0, respectively. CI: 95%, *p* = 0.005 (Figure 3). BC did not show any difference when compared to the other groups (Figure 4).

## 4. Discussion

Many different methods to place the sealer within the root canal have been described in the literature [14]. Some studies reported that all techniques have similar results [15,16,17], while others, like Kahn et al. [16,18], concluded that Max-i-probe or lentulo spirals had shown the best results, followed by ultrasonic/sonic files. A recent publication on this topic considered that different obturation techniques associated with different sealer’s placement could produce different results related to the outcome [11]. In the present study, we analyzed the influence of the activation of the sealer with sonic activation (EndoActivator) on different obturation techniques, and in concordance with a recent research conducted by Wiesse et al. [19], activation of the sealer with this device did not produce a statistically significant effect when comparing the different groups. In agreement with Wiesse et al. [19], even if sonic activation causes a hydrodynamic phenomenon in irrigation solutions [20], it does not seem to occur with root canal sealers, possibly due to the high density of the sealer reducing the effectiveness of the EndoActivator at a high amplitude and at a low frequency (1 to 6 kHz) [21]. On the other hand, these authors found a positive effect of the use of ultrasonic activation for this purpose as demonstrated in a previous investigation [22]. Nevertheless, the use of ultrasonic activation within curved root canals must be considered with care as uncontrolled removal of dentin could occur [23]. Only one research has found that EndoActivator performed well when compared to an ultrasonic device and a non-activation group [24], reporting a greater penetration of sealer into simulated accessory canals. Clinically, the major implication is that applying the sealer directly in the canal by coating the lumen with paper points or files would be sufficient without the need for ultrasonic activation. This is particularly relevant clinically when utilizing carrier-based techniques that do not allow for coating the gutta-percha, which is heated extra-orally.

The Thermafil Plus system has been studied widely in the literature; nevertheless, there is not much information about GuttaCore and GuttaFusion systems [5,25]. The core of these systems consists of a “thermostable semi-rigid cross-linked gutta-percha”, which resists the temperature generated by the oven designed for these materials [26]. Besides this, re-treatment of both systems is easier and faster [27]. According to the manufacturer, both systems are similar, and they only differ in grip and core color. Therefore, we compared these core-based systems with each other, as previously done by Schäfer et al. [25]. In the present investigation, good comparable results were observed for both filling systems. Nevertheless, the precursor of these systems, Thermafil, scored a significantly lower percentage of gutta-percha filled areas and a higher percentage of voids at the apical level, and it was less predictable at the other levels studied. A possible explanation could be the use of an obturator of different size for the two cross-linked gutta-percha systems (size #30 for Thermafil and size #25 for both cross-linked gutta-percha systems [5]), as per manufacturer recommendation, for the final size of preparation used in this study. Neuhaus et al. [28] did not find any differences between Thermafil and GuttaFusion, but the size of obturators used in their research is unclear. Marciano et al. [29] discuss in their publication that there is a variety of anatomical irregularities that could affect the results of studies done with natural teeth, even if natural teeth are able to reproduce a clinical situation in a better way. This should be considered when interpreting the results. The different composition of the core (gutta-percha) of the new core-based systems could suggest that the heat of the oven could affect the performance of these systems and produce a lower pressure during the insertion into the canal. However, in the present study, as well as in a precedent study from Neuhaus et al. [28], this result was not found. The use of carrier-based approach is now clinically accepted and leads to major advantages, especially when dealing with curved canals where the carrier compaction forces are equally distributed along the whole length of the root canal, as compared with pluggers that cannot be used past abrupt curvature. The absence of voids in the apical third are relevant in leading to relapse of endodontic infections [30].

The EndoSequence BC sealer has been shown to improve some properties of AH Plus sealer [7]. In research conducted by Zhou et al. [6], AH Plus sealer exhibited shrinkage, while a slight expansion was measured with the EndoSequence BC sealer. These results could suggest that EndoSequence BC sealer could have a better performance regarding to occupation of voids and infill of dentinal tubules. Nevertheless, and in concordance with Celikten et al. [31], our results do not show a better performance of this material from this point of view. The use of bioceramic sealer appears to be promising in a situation where long-sustained microbial activity may be required, as these materials can lead to the development of an alkaline environment for a long time, tackling persisting bacteria present in the dentinal tubules or in the main lumen [32].

CLSM was used in the present investigation, to assess the root-canal-filling quality. CLSM has been defined as a versatile method [33,34], as it gives adequate information on the adaptation and/or distribution of the sealer within the root canal system and dentinal tubules. Performed with fluorescent dyes, such as rhodamine-B, it is an appropriate method for the evaluation of materials with different composition [35], giving adequate information about the root canal obturation quality, as the detection of voids is suitable with this method [36]. Filling materials within the canal or residual obturation material remnants after desobturation have been previously evaluated with this method [29,37].

Assessment with CLMS minimize artifacts as it does not need a special treatment for the samples (including management in normal environmental conditions) [34].

In the present investigation, optical microscopy images were also used for the evaluation, in order to create a visual reference to match the confocal images previously obtained. Conversely, the CLMS evaluation cannot give volumetric information. Micro-CT has shown to be superior in this sense; nevertheless, as sealers could be radiopaque, leading to backscattering, the voids could be underestimated with micro-CT’s being less sensitive as compared with the sectioning methods [10,38].

Even though an obturation system produces a high percentage of gutta-percha filled areas, occurrence of voids should also be assessed. Detection of voids is an important parameter for evaluating the quality of a root canal obturation system [11]. In the present study, this parameter was an indirect measure, as it is equal to the subtraction of the PGFA and PSFA from the canal area percentage value. The presence of voids may be dependent on several factors, such as the type of sealer used, the method of application and the obturation technique utilized. The present in vitro study has a direct applicability to the clinical scenario, as it revealed that the use of ultrasonic activation did not play a significant role in reducing the number of voids; on the other hand, cross-linked gutta-percha provided a significantly more homogenous root canal filling, as compared with the cold-lateral-compaction approach. Future research should evaluate the promptness of reinfection of the root canal system based on different techniques used in the absence of coronal seal. Finally, the contribution of tubular infection acting as a reservoir of pathogens and leading to relapse of endodontic infection should be determined.

## 5. Conclusions

Within the limitations of this study, gutta-percha area, sealer area and voids were dependent on the obturation system used, exhibiting better results for cross-linked gutta-percha systems. GuttaCore and GuttaFusion produced very homogenous obturations with high PGFA and a low incidence of voids at coronal and apical levels, respectively. Sonic activation of the sealer does not improve the root-canal-filling quality. Additional clinical research is needed to determine if other variables may affect the root-canal-filling quality. Clinical implications of a material with less voids are apparent, especially in cases where coronal seal deficiency may lead to recontamination of the root canal space. The presence of voids represents a prompt route of reinfection and a niche where newly formed biofilm can develop. Carrier based obturation systems can produce a homogenous obturation that is free of voids.

## Figures and Tables

**Table 1:** Mean and Standard Deviation of percentage of gutta-percha filled areas (PGFA), percentage of cement filled areas (PSFA) and percentage of void areas (PVA) at 3 mm, 6 mm and 9 mm. GP, gutta-percha; BC, EndoSequence BC sealer; GF, GuttaFusion; TH, Thermafil; GC, GuttaCore; CL, cold lateral compaction.

	GROUP	GP % (Mean-SD)	Sealer % (Mean-SD)	Voids % (Mean-SD)	GP % (Mean-SD)	Sealer % (Mean-SD)	Voids % (Mean-SD)	GP % (Mean-SD)	Sealer % (Mean-SD)	Voids % (Mean-SD)
Distance from the apex		3 mm	3 mm	3 mm	6 mm	6 mm	6 mm	9 mm	9 mm	9 mm
No Activation	BC	69.0–12.2	29.8–11.5	0.5–1.0	61.9 14.2	35.7–15.1	0.6–1.4	56.6–12	41.9–11.4	2.5–3.3
	CL	75.4–16.2	23.7–15.2	1.5–2.5	79.1–11.0	18.1–10.3	1.4–1.5	82.0–10.9	15.6–10.0	2.6–2.5
	GC	76.1–33.6	23.1–33	1.0–1.6	88.5–16.7	10.3- 15.7	0.3–1.0	91.5–4.1	7.9–3.5	0.6–0.8
	GF	90.7–7.4	8.9–7.6	0.1–0.2	92.5–7.9	6.2–5.9	0.8–1.9	91.7–5.2	7.5–5.0	1.7–2.7
	TH	54.4–46.1	43.3–43.5	2.1–5.3	94.3–5.4	4.8–5.1	0.3–0.6	91.9–7.0	6.8–6.2	1.3–2.0
Activation	BC	60.1–12.4	39.3–12.1	1.2–1.9	60.2–12.9	39.3–12.7	2.5–4.2	56.7–11.4	40.8–11.9	1.5–4.3
	CL	72.1–15.2	26.5–14.8	0.9–2.3	80.6–7.5	18.1–7.2	2.8–3.3	81.7–13.1	15.8–11.8	2.4–1.8
	GC	73.0–34.0	26–32.7	0.8–1.3	83.7–27.7	16.0–27.0	1.2–2.7	91.3–4.9	8.0–4.8	0.5–1.0
	GF	91.1–7.1	8.8–7	0.3–0.9	92.9–5.4	6.3–4.6	1.3–2.8	91.7–6.8	6.6–5.5	0.8–1.8
	TH	77.8–32.1	20.2–28.2	2.4–4.5	94.0–5.5	5.7–5.3	1.0–1.8	92.7–3.9	6.0–3.4	1.3–1.6

## Data Availability

Data is contained within the article.
